# Development and content validation of a patient-reported endometriosis pain daily diary

**DOI:** 10.1186/s12955-017-0819-1

**Published:** 2018-01-04

**Authors:** Floortje E. van Nooten, Jennifer Cline, Celeste A. Elash, Jean Paty, Matthew Reaney

**Affiliations:** 1 Astellas Pharma, Leiden, the Netherlands; 2 PinneyAssociates, 201 N. Craig street, suite 320, Pittsburgh, PA 15213 USA; 3 ERT, 225 W Station Square Dr, Ste 220, Pittsburgh, PA 15219 USA; 4 Quintiles IMS Incorporated, One IMS Way, Plymouth Meeting, PA 19462 USA; 5 ERT, Peterborough, PE2 6FZ UK

**Keywords:** Endometriosis, Patient-reported outcome measures, Dysmenorrhea, Pelvic pain

## Abstract

**Background:**

Endometriosis is a common gynecological disorder that causes inflammation and pelvic pain. Endometriosis-related pain is best captured with patient-reported outcome (PRO) measures, however, assessment of endometriosis-related pain in clinical trials has been difficult in the absence of a reliable and valid PRO instrument. We describe the development of the Endometriosis Pain Daily Diary (EPDD), an electronic PRO developed as a survey instrument to assess endometriosis-related pain and its impact on patients’ lives.

**Methods:**

The EPDD was initially developed on the basis of an existing Endometriosis Pain and Bleeding Diary, a targeted review of relevant literature, clinical expert interviews, and open-ended (concept elicitation) patient interviews in the United States (US) and Japan which captured patients’ experience with endometriosis. Cognitive interviews of patients with endometriosis were conducted to evaluate patient comprehension of the EPDD items. A conceptual model of endometriosis was developed, and meetings with US and European regulatory authorities provided feedback for validating the EPDD in the context of clinical trials. Translatability assessments of the EPDD were conducted to confirm its appropriate interpretation and ease of completion across 17 languages.

**Results:**

The iterative development progressed through three versions of the instrument. The EPDDv1 included 18 items relating to dysmenorrhea/pelvic pain, dyspareunia and sexual activity, bleeding, hot flashes, daily activities, and use of rescue medication. The EPDDv2 was a larger 43-item survey tested in cognitive interviews and subsequently revised to yield the current 11-item EPDDv3, consisting of five core items relating to dysmenorrhea, non-menstrual pelvic pain, and dyspareunia, and six extension items relating to sexual activity, daily activities, and use of rescue medication.

**Conclusions:**

The EPDD is a PRO for the evaluation of endometriosis-related pain and its associated impacts on patients’ lives. The EPDD represents an important step in providing a PRO that is relevant to patients with endometriosis-related pain in the context of a clinical study setting (ie, fit-for-purpose), designed to evaluate pain associated with endometriosis, including regulatory agency support for its further exploration in clinical trials.

## Background

Endometriosis is a common gynecological disorder characterized by the presence of endometrial glands and tissue outside of the uterus, resulting in chronic inflammation, dysmenorrhea, dyspareunia, and chronic pelvic pain [1]. The prevalence of endometriosis ranges from 6 to 10% in the worldwide female population [2], and has been shown to be associated with dysmenorrhea, dyspareunia, and chronic pain. Women with endometriosis experience absenteeism and reduced overall work productivity as a result of their condition, and have demonstrated reduced quality of life scores on the Short Form-36 version 2. Additionally, among women in a relationship, endometriosis can negatively impact partner relationships [3, 4].

Endometriosis-related pain and its impact on patients’ lives are best captured through the use of direct reports from patients regarding their experience, that is, patient-reported outcome (PRO) measures. PROs are widely used in evaluating pain; three PROs are commonly used in studies of endometriosis. The Composite Pelvic Signs and Symptoms Score (CPSSS) is a modified version of the Biberoglu and Behrman Scale [5] and measures dysmenorrhea, dyspareunia, non-menstrual pelvic pain (NMPP), pelvic tenderness, and pelvic induration. This scale was developed on the basis of physician opinion, without specific input from patients, and has a 28-day recall period [6]. Given that responses to PRO items are likely to be influenced by the patient’s state at the time of recall, the United States (US) Food and Drug Administration (FDA) PRO Guidance for Industry recommends, in alignment with good measurement practices [7, 8], that PROs have a recall period that is appropriate to capture the patient’s current or recent state [9]. Additionally, patient data are recorded by the physician and thus the CPSSS is not a direct PRO. The Endometriosis Health Profile (EHP) is a PRO instrument that measures the impact of endometriosis on patients’ lives; specifically, the EHP measures pain, control and powerlessness, social support, emotional well-being, and self-image [10–13]. The EHP was designed to measure the impact of endometriosis rather than specific symptoms and has a 4-week recall period. The Endometriosis Pain and Bleeding Diary (EPBD) [14] was developed to measure endometriosis symptoms. Although the EPBD was developed on the basis of both patient and physician input, and designed to collect information daily, the instrument’s development was discontinued prior to establishing that it was fit-for-purpose.

Thus, the CPSSS, EHP, and EPBD have their limitations as a fit-for-purpose tool for evaluating endometriosis-related pain in the context of supporting labeling claims of a treatment benefit, as outlined in the US FDA PRO Guidance for Industry [9]. Therefore, we have developed the Endometriosis Pain Daily Diary (EPDD) as a fit-for-purpose PRO instrument to assess pain and its impact on patients’ lives in clinical trials for women with endometriosis-related pain.

Given its development process, the EPBD was an appropriate starting place for the EPDD. The FDA guidance emphasizes the importance of demonstrating evidence of the relevance (ie, content validity) of the PRO instrument to the target patient population, and ensuring that the population studied in the PRO instrument development and documentation process is comparable with that in the clinical study setting in which the instrument is to be used (ie, that the instrument is fit-for-purpose).

This paper describes the initial steps of the development of the EPDD. Specifically, this paper describes the process undertaken for establishing content validity of the EPDD, providing evidence that it measures relevant concepts and experiences relating to pain associated with endometriosis, is homogenously interpretable, and is easily comprehendible. The processes described herein represent the first steps of development of an instrument that is ‘fit-for-purpose’.

## Methods

**Fig. 1:**
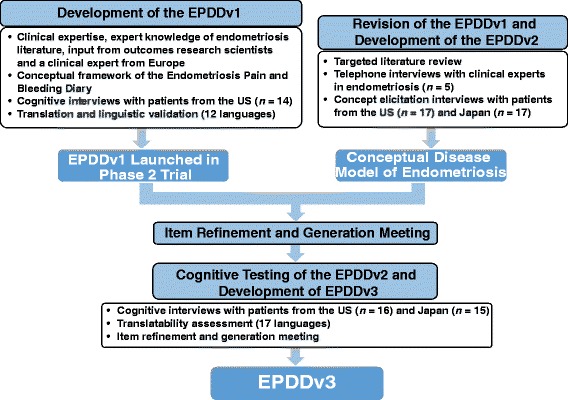
Development Process of the EPDD

The EPDD was developed through a series of procedures, including the development of an evidence-supported conceptual model of disease, all of which followed regulatory guidance set forth by the European Medicines Agency’s (EMA) Committee for Medicinal Products for Human Use (CHMP) reflection paper on the use of health-related quality of life measures in the evaluation of medicinal products [15] and the FDA PRO Guidance for Industry [9]. The recommendations in these regulatory guidelines are aligned with good instrument development practices [7, 8]. The development process described herein encompasses all procedures that took place in the development of the EPDD as a content-valid PRO designed for the purpose of assessing treatment response in clinical trials (Fig. 1).

The EPDD was designed to be administered using an electronic platform, asking patients to record their endometriosis-related pain experiences over the previous 24 h. To date, the EPDD has been administered using a small hand-held electronic device that queries the patient each evening for a recording. The device allows patient compliance to be tracked and is designed to minimize incomplete records, as the programming does not allow patients to skip questions.

### Development of the EPDDv1

The initial version of the EPDD (EPDDv1) was developed on the basis of the conceptual framework of the existing Endometriosis Pain and Bleeding Diary (EPBD) [14], available literature on endometriosis, and input from outcomes research scientists and a clinical expert from Europe (Fig. 1). In order for the EPDD to be implemented in a phase 2 clinical trial of endometriosis (TERRA; NCT01767090), cognitive interviews were conducted to test for patient comprehension of the draft items, language, instructions, and response options. Specifically, the cognitive interviews were designed to gain the patient’s perception of the survey items (ie, what the patient believes the question is asking) and comprehension of terms (ie, what specific words and phrases mean to the patient) [16]. Semi-structured, individual, face-to-face interviews were conducted with 14 women from the US with surgically confirmed endometriosis and consisted of patients completing the EPDDv1 and answering questions relating to the intent of the questions and the interpretation of specific terms. Interviewers were research scientists with training and extensive experience conducting qualitative interviews with patients across a number of therapeutic indications including endometriosis and other areas of women’s health. In preparation for these studies, the interviewers reviewed the content and purpose of semi-structured interview guide questions with the authors and conducted practice interviews with each other to become familiar with interview content and flow. Near the end of the interview, the patient’s acceptance of the electronic platform was assessed with a brief series of questions assessing ease of use, ease of response selection, acceptability of the electronic platform and any problems using the electronic platform. The information obtained from these exercises was used to inform the finalization of the EPDDv1. The EPDDv1 was then translated and linguistically validated in 12 languages (including English) to ensure conceptual equivalency of the survey items in the 10 countries that enrolled patients for the global phase 2 TERRA study. Prior to translation, all language was reviewed for any potential modification that would be required to ensure that the conceptual basis of each question would be retained. The translation and linguistic validation followed internationally accepted standards set forth by the International Society for Pharmacoeconomics and Outcomes Research and consisted of seven processes: 1) a dual forward translation was performed by two independent native-speaking translators; 2) a third native-speaking linguist assessed the translations and reconciled any discrepancies between the two translations; 3) a native speaker with proficiency in English performed a back translation to ensure that the forward translation was conceptually equivalent to the original; 4) a medical review was conducted to ensure that the survey items were appropriate for a clinical setting; 5) cognitive testing of the EPDDv1 was conducted on a small group of relevant patients with endometriosis in order to test alternative wording and to check understandability, interpretation, and cultural relevance of the translation; 6) a review of cognitive testing results compared the patients’ interpretation of the translation with the original version to highlight and amend discrepancies and finalize the translation; 7) the final step was proofreading and final review of the translation [17].

### Revision of the EPDDv1 and development of the EPDDv2

Following the launch of the phase 2 TERRA study that employed the EPDDv1, the tool was further evaluated for content validity using updated literature reviews, clinician interviews, and additional, semi-structured patient interviews. This three-step process was designed to evaluate whether items needed to be modified or deleted, or if additional items should be added to the EPDDv1. The following three steps (Fig. 1) were:

- A targeted literature review to identify the most important and relevant symptoms and impacts related to endometriosis. The MEDLINE database was searched for human studies published in English; search terms included targeted phrases such as ‘endometriosis sign’, ‘endometriosis symptom’, ‘endometriosis impact’, ‘endometriosis’ and ‘quality of life’, ‘Activities of Daily Living (ADL)’, ‘interview’, and ‘focus group’.
- Individual interviews with five clinical experts in endometriosis to collect further input regarding important and relevant health concepts (ie, signs, symptoms, side-effects, impacts) for women with endometriosis.
- Concept elicitation interviews with adult women in the US and Japan to identify and confirm the symptom and impact concepts from the EPDDv1 that were the most important and relevant to women with endometriosis-related pain. Patients from the US (*n* = 17) were recruited if they were aged 18–45 years and had surgically confirmed diagnosis of endometriosis within 5 years. Patients from Japan (*n* = 17) were recruited if they were aged 20–45 years with a diagnosis of endometriosis; because surgical diagnosis is uncommon in Japan, surgical diagnosis was not required if diagnosis was confirmed by a blood test, internal examination, or a magnetic resonance imaging or computed tomography scan. During the interviews, patients were first asked to spontaneously report any symptoms they experienced as a result of endometriosis, and thereafter were asked if they experienced any specific symptoms from a predefined list of symptoms known to be associated with endometriosis (based on literature and expert input).

The digital audio files from the patient concept elicitation interviews were transcribed into document files and loaded into the ATLAS.ti (version 5.0) software program for coding [18]. Data coding methodology followed a three-step process. First, the coder identified each incidence of a concept expression in the transcript text. Second, the coder highlighted the actual patient quote as the assigned code name and then matched the tagged text to a code stem from the coding framework, allowing it to be grouped with other codes of similar content related to the project’s objectives. If a code stem did not exist in the coding framework, a new one was developed and the coding framework was expanded; any code stems not used were removed when the coding dictionary was finalized. Third, the final coding dictionary was the result of the full set of concept codes identified in the patient transcripts, organized by the overall structure of the coding framework so the results could be related to the project objectives. All descriptive data from the screening form were entered into SPSS (version 11.5) to generate tables of descriptive statistics.

Information obtained from these exercises resulted in the development of a conceptual disease model of endometriosis. This conceptual disease model served to facilitate an item refinement and generation meeting, during which the EPDDv1 was updated to include relevant findings from the literature review and patient and expert interviews. The outcome of the item refinement and generation meeting was the EPDDv2.

### Cognitive testing of the EPDDv2 and development of the EPDDv3

The EPDDv2 was tested in additional cognitive interviews with patients. Patients with endometriosis from the US (*n* = 16) and Japan (*n* = 15) were identified via clinical record review (US) and patient self-reporting (Japan), and subsequently recruited and screened at three US centers (Spokane, WA; Seattle, WA; New Orleans, LA) and one market research firm in Tokyo, Japan, to participate in the cognitive interviews. Patients completed the EPDDv2 and participated in face-to-face individual semi-structured interviews.

Following minor refinements made during and following the cognitive interviews, a translatability assessment was conducted to identify and rectify any major conceptual issues to ensure appropriate interpretation of the EPDDv3 and address any potential concerns regarding cross-cultural adaptation before its potential use in pivotal trials. Independent translators assessed the translatability of the 11-item EPDDv3 in 17 languages (Bulgarian, Dutch, English, French, German, Hungarian, Italian, Korean, Polish, Portuguese, Romanian, Russian, Simplified Chinese, Traditional Chinese, and Spanish [for Spain, US, and Argentina]) among 19 countries. When countries/languages are selected for the pivotal trials, the full translation and linguistic validation process would be conducted for each language. Overall, results from the translatability assessment demonstrated that the EPDDv3 is a robust tool and has utility among all translated languages. No significant issues were identified. Furthermore, additional regulatory feedback was obtained from the FDA and the EMA and a second item refinement and generation meeting was held during which the current version of the EPDD (EPDDv3) was developed.

## Results

### Development of the EPDDv1

The EPDDv1 included 18 items in six categories: pelvic pain (including dysmenorrhea and NMPP), dyspareunia and sexual activity, bleeding, hot flashes, daily activities, and rescue medication/protection. The EPDDv1 was developed to be administered electronically with a 24-h recall period. In the phase 2 TERRA trial, it was implemented with a small hand-held device.

### Semi-structured cognitive interviews for EPDDv1

A total of 14 patients from the US participated in the face-to-face, semi-structured cognitive interviews in three waves, with 4–5 patients in each wave. During the first wave, wording modifications were made to two instructions and 7 items of the EPDDv1. During the second wave, wording modifications were made to one item. No modifications were made during the third wave. Demographic data are presented in Table 1. Results from the Technology Ease of Use Questionnaire indicated that the participating patients found the electronic hand-held device displaying the EPDDv1 to be easy to use and well understood (Table 2).

**Table 1:** Demographics for US Patient Cognitive Interviews for the EPDDv1

Characteristic		US (*N* = 14)
Age, years	Mean (SD)	34.1 (6.8)
Highest level of education completed	High school	3 (21.4)
	Some college	6 (42.9)
	Bachelor’s degree	2 (14.3)
	Graduate or professional school	3 (21.4)
Ethnicity	White/Caucasian (non-Hispanic)	13 (92.9)
	White/Caucasian (Hispanic)	0
	Black/African American	1 (7.1)
	Hispanic or Latino	0
General health	Excellent	1 (7.1)
	Very good	6 (42.9)
	Good	5 (35.7)
	Fair	2 (14.3)
	Poor	0
Over past month, endometriosis-related pain severity *during menstruation* (0 = no pain to 10 = worst pain imaginable)	Mean (SD)	7.2 (1.7)
Over past month, endometriosis-related pain severity *not during menstruation* (0 = no pain to 10 = worst pain imaginable)	Mean (SD)	5.8 (1.6)

All data are presented as n (%) unless otherwise noted

**Table 2:** Technology Ease of Use Questionnaire – Results from US Patient Cognitive Interviews for the EPDDv1

Questionnaire Item	Response Item	*n* (%)
How easy/difficult did you find using the electronic questionnaire?	Very easy	6 (42.9)
	Quite easy	3 (21.4)
	Very difficult	–
	Missing^a^	5 (35.7)
The choices that were there to use when I answered the questions were:	Easy to read on the screen, no problem choosing my response	8 (57.1)
	A little difficult to read on the screen, and I had some difficulty in choosing my response	1 (7.1)
	Missing^a^	5 (35.7)
Overall, did you find the electronic questionnaire acceptable to use?	Yes	9 (64.3)
	No	–
	Missing^a^	5 (35.7)

^a^The Technology Ease of Use Questionnaire was inadvertently not administered to one wave of cognitive interviews (5 patients)

## Revision of the EPDDv1; development of EPDDv2

### Targeted literature review

A total of 30 peer-reviewed articles were retrieved from the search; seven articles contained qualitative data. Five types of pain were identified, and bleeding and gastrointestinal disturbance were also highlighted as being related to endometriosis.

### Clinical expert interviews

Five clinical experts were recruited from Europe (United Kingdom, *n* = 1; Germany, *n* = 2) and Japan (*n* = 2) and completed a semi-structured interview. Interviews with the Japanese experts were conducted in English via typed text to minimize language misinterpretation. All five clinical experts highlighted the key symptom of endometriosis as pain; specifically, pain during menstruation, NMPP, pain during and after sexual intercourse, and pain during defecation. Pain during or after sexual intercourse was more associated with ‘deep’ pain rather than ‘superficial’ pain. A less common symptom not related to pain was bleeding. All clinical experts stated that a primary concern related to endometriosis is infertility. The most common impacts on patients’ daily lives, as perceived by clinical experts, were role functioning, especially attendance at work and school, performance of daily activities, sexual function, and partner relationships; missed work/school was consistently reported as having the greatest impact.

### Concept elicitation interviews

A total of 34 female patients were recruited from six US clinical sites and one Japanese market research firm; demographic data are presented in Table 3.

**Table 3:** Demographics for US and Japanese Patient Concept Elicitation Interviews

Characteristic		US (*N* = 17)	Japan (*N* = 17)
Age, years	Mean (SD)	30.5 (6.6)	41.1 (5.0)
Marital status	Married	5 (29.4)	13 (76.5)
	Living with partner	4 (23.5)	0
	Divorced	2 (11.7)	2 (11.8)
	Never married	6 (35.2)	2 (11.8)
Highest level of education completed	High school	1 (5.9)	1 (5.9)
	Some college	8 (47.1)	8 (47.1)
	Bachelor’s degree	3 (17.6)	7 (41.2)
	Graduate or professional school	5 (29.4)	1 (5.9)
Current employment status	Not employed outside of home	2 (11.8)	2 (11.8)
	Employed full-time	8 (47.1)	6 (35.3)
	Employed part-time	3 (17.6)	1 (5.9)
	Retired	0	1 (5.9)
	Not employed	4 (23.5)	7 (41.2)
Ethnicity	White/Caucasian (non-Hispanic)	14 (82.4)	–
	White/Caucasian (Hispanic)	2 (11.7)	–
	Hispanic or Latino	1 (5.9)	–
General health	Excellent	0	1 (5.9)
	Very good	6 (35.2)	4 (23.5)
	Good	8 (47.0)	8 (47.1)
	Fair	3 (17.6)	4 (23.5)
Over past month, endometriosis-related pain severity *during menstruation* (0 = no pain to 10 = worst pain imaginable)	Mean (SD)	7.3 (1.5)	6.9 (1.9)
	Median	7.0	7.0
	Range	4–10	5–10
Over past month, endometriosis-related pain severity *not during menstruation* (0 = no pain to 10 = worst pain imaginable)	Mean (SD)	5.9 (1.4)	3.2 (2.3)
	Median	5.0	3.0
	Range	4–9	0–7

All data are presented as n (%) unless otherwise noted

Concepts that are spontaneously expressed during the open-ended portions of the interview can be considered to be of higher importance or relevance to the patient than those expressed as a result of probing by the interviewer. Among US patients, the most common symptoms associated with endometriosis were ‘pain during menstruation’ and ‘NMPP’, both spontaneously reported by 82.4% (14 of 17) of patients who reported these symptoms (Table 4). Among Japanese patients, the most common symptoms associated with endometriosis were the same, with ‘pain during menstruation’ and ‘NMPP’ spontaneously reported by 100% (17 of 17) and 76.5% (13 of 17) of patients who reported these symptoms, respectively (Table 4).

**Table 4:** Symptoms Associated With Endometriosis

Symptom	Spontaneous		Probed		Not Affected		Not reported	
	US	Japan	US	Japan	US	Japan	US	Japan
Pain during menstruation	14 (82.4)	17 (100)	2 (11.7)	–	–	–	1 (5.9)	–
NMPP	14 (82.4)	13 (76.5)	2 (11.7)	2 (11.8)	–	1 (5.9)	1 (5.9)	1 (5.9)
Superficial vaginal pain during sexual intercourse	1 (5.9)	1 (5.9)		–	–	15 (88.2)	16 (94.1)	1 (5.9)
Deep vaginal pain during sexual intercourse	1 (5.9)	–	2 (11.7)	9 (52.9)	10 (58.8)	7 (41.2)	4 (23.5)	1 (5.9)
Superficial vaginal pain after sexual intercourse	1 (5.9)	–	1 (5.9)	1 (5.9)	–	15 (88.2)	15 (88.2)	1 (5.9)
Deep vaginal pain after sexual intercourse	3 (17.6)	1 (5.9)	7 (41.2)	4 (23.5)	3 (17.6)	10 (58.8)	4 (23.5)	2 (11.8)
Other symptoms^a^	10 (58.8)	3 (17.6)	NA	–	–	–	7 (41.2)	14 (82.4)

All data are presented as n (%)

NMPP, non-menstrual pelvic pain

^a^Other symptoms spontaneously offered by US patients: ache in lower back; back aches; bloating; bowel; dull achy; exhaustion; headache; heavy menstrual flow; heavy periods; hot flashes/night sweats; lower back ache; nausea; ovary in pain before sex; pain after sexual intercourse in pelvic area; painful bowel movements; rectal bleeding; sharp stabbing pain left lower abdomen; stabbing pain around right ovary; vomiting; and warming sensation

Other symptoms spontaneously offered by Japanese patients: watching TV suddenly have pain (pain at all times); heaviness lie on side lower back (stomach); heavy bleeding/anemia/dizzy/squeezing pain in stomach-used to be around back feel that uterus being pulled down (clots)

The most common impacts associated with endometriosis were ‘problems doing physical activities’, spontaneously reported by 70.6% of US patients (*n* = 17) and 64.7% of Japanese patients (*n* = 17), and ‘having problems at work’, spontaneously reported by 58.8% of US patients and 41.2% of Japanese patients. Japanese patients also commonly reported ‘dropping other daily activities’ (41.2%) (Table 5).

**Table 5:** Impacts Associated With Endometriosis

Symptom	Spontaneous		Probed		Not Affected		Missing	
	US	Japan	US	Japan	US	Japan	US	Japan
Problems doing physical activities	12 (70.6)	11 (64.7)	4 (23.5)	2 (11.8)	1 (5.9)	1 (5.9)	–	3 (17.6)
Affecting other daily activities	6 (35.3)	7 (41.2)	7 (41.2)	7 (41.2)	4 (23.5)	2 (11.8)	–	1 (5.9)
Experience difficulties during or after sexual intercourse (changes in relationship with partner)	8 (47.1)	7 (41.2)	5 (29.4)	5 (29.4)	4 (23.5)	4 (23.5)	–	1 (5.9)
Doing fewer social activities (seeing friends and acquaintances less often)	9 (52.9)	2 (11.8)	7 (41.2)	2 (11.8)	1 (5.9)	12 (70.6)	–	1 (5.9)
Having problems doing your daily chores at home (cleaning, cooking, house maintenance)	3 (17.6)	4 (23.5)	10 (58.8)	10 (58.8)	4 (23.5)	2 (11.8)	–	1 (5.9)
Having problems at work (with people or with getting your work completed)	10 (58.8)	6 (35.3)	3 (17.6)	8 (47.1)	4 (23.5)	2 (11.8)	–	1 (5.9)
Other impacts^a^	12 (70.6)	10 (58.8)	NA	–	–	–	5 (29.4)	7 (41.2)

All data are presented as n (%)

^a^Other impacts spontaneously offered by US patients: avoid work out; competitive runner; depression; family life (taking care of her daughter); fatigue; fertility – ability to have children; having another child; irritable relationship; lifting children; limit time away from home; low libido; medical issue stomach; school; sleep; sleep a lot; thinking about having hysterectomy; and tiredness

Other impacts spontaneously offered by Japanese patients: bowel movement; [have] to stay in bed for 2 days; can’t [fall] asleep; financial travel plan; avoid those times (intercourse pain, ovulation period, bowel movement pain); [have] to take a day off in school (past); lying down (sideways); lying down; have to rest; leave her alone not to be disturbed; not able to sit; prefer to stay in bed; cannot keep standing especially on Day 1

### Integrated findings

Table 6 summarizes the findings from all three sources of evidence, the literature review, and the clinician and patient interviews. The findings show that there was consistency from all three sources with regard to types of pain, but less overlap with other symptoms and their impact on patients’ lives.

**Table 6:** Key Concepts Identified in Literature, and Expert and Patient Interviews

Concept	Literature Review	Expert Interviews	Patient Interviews
Pain during menstruation	✓	✓	✓
Pain during/after sexual intercourse	✓	✓	✓
Pain unrelated to sex and/or menstruation (pelvic, back, general, headache)	✓	✓	✓
Pain during defecation	✓	✓	✓
Pain during urination	✓		
Other non-pain symptoms (bloating, dizziness, nausea, vomiting)			✓
Infertility		✓	
Bleeding (during menstruation, sex, or irregular)	✓		✓
Gastrointestinal disturbance (diarrhea, constipation, flatulence)	✓		
Work/school limitations	✓	✓	✓
Sexual activity limitations	✓	✓	✓
Difficulty doing daily activities	✓	✓	✓
Difficulty doing leisure activities	✓		✓
Physical activity limitations	✓		✓
Social/lifestyle limitations	✓		✓
Relationships affected	✓	✓	✓
Emotional health	✓	✓	✓
Sleep difficulties			✓
Low energy difficulties			✓
Coping behaviors			✓

### Conceptual model of disease

**Fig. 2:**
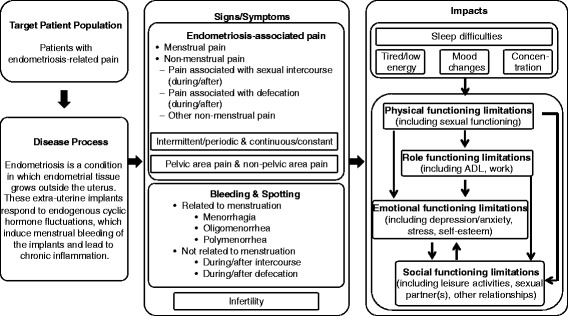
Conceptual Model of Endometriosis Note: Symptoms not related to pain (eg, bleeding, hot flashes, infertility) and impacts not expected to change week by week were not included in the EPDDv3 based on regulatory feedback and additional decisions made in a final item refinement and generation meeting held prior to the development of EPDDv3. ADL, activities of daily living

Analysis of data obtained from the concept evaluation exercises (ie, literature review, expert interviews, and patient interviews) informed a conceptual model of disease (Fig. 2), which served to facilitate the item refinement and generation meeting. The conceptual model of endometriosis includes symptom concepts related to pain and vaginal bleeding during defecation and gastrointestinal disturbances, and expands on impacts relating to sleep disturbances, physical functioning limitations, and social functioning limitations. During the item refinement and generation meeting, the EPDDv2 was developed based on this conceptual model to be fully comprehensive. The EPDDv2 consisted of 43 items comprising revised iterations of the original 18 items of the EPDDv1 and an additional 24 items related to pain/bleeding during defecation, gastrointestinal disturbance, and difficulties with functioning and/or sleep.

### Cognitive testing of the EPDDv2; development of the EPDDv3

Patients were recruited and screened at three US centers (Spokane, WA; Seattle, WA; New Orleans, LA) and through a market research firm in Tokyo, Japan. The cognitive interviews were conducted in four waves in the US and three waves in Japan. Patient demographics are presented in Table 7.

**Table 7:** Demographics for US and Japanese Patient Cognitive Interviews for the EPDDv2

Characteristic		US (*N* = 16)	Japan (*N* = 15)
Age, years	Mean (SD)	33.2 (6.3)	34.9 (6.4)
	Median	33.0	35.0
	Range	24–43	24–44
Highest level of education completed	High school	–	7 (46.7%)
	Some college	9 (56.3%)	3 (20.0%)
	Bachelor’s degree	5 (31.3%)	5 (33.3%)
	Graduate or professional school	2 (12.5%)	–
Worst endometriosis-related pain during menstruation: (0 = no pain to 10 = worst pain imaginable)	Mean (SD)	8.0 (1.2)	7.5 (1.6)
	Median	8.0	8.0
	Range	6–10	4–10
Worst endometriosis-related pain when not menstruating: (0 = no pain to 10 = worst pain imaginable)	Mean (SD)	5.6 (1.7)	4.8 (2.9)
	Median	5.5	5.0
	Range	2–9	0–9

The EPDDv2 items were tested in three waves of cognitive interviews in the US, and wording modifications were made to items after each wave. During Wave 1 (US), wording modifications were made to three items pertaining to pain, sexual intercourse and pain, and bleeding; no items were added or removed. During Wave 2 (US), wording modifications were made to one item, and alternate questions for nine items were proposed and tested in Wave 3. During Wave 3 (US), one item was removed from the survey, and an alternate question was proposed for one other item for further testing.

The EPDDv2 was then translated to Japanese and tested in two waves of interviews in Japan. The Japanese translation was slightly modified after each wave to ensure the accuracy and understandability of the language used. Based on Japanese patient feedback, translation modifications were necessary for the phrases ‘deep pain or penetration’, ‘spotting’, ‘social activities’, sexual ‘interest’, and ‘during the past 24 h’, which are not common terminology in Japan. Four items were evaluated and found to be better understood by patients and subsequently replaced the original items.

Following the two waves of interviews in Japan, and based on feedback from the FDA and EMA regarding the length of the survey, the EPDDv2 was reduced from 43 to 11 items consisting of five core items relating to dysmenorrhea, NMPP, and dyspareunia, and six extended items relating to sexual activity, daily activities, and use of rescue medication, yielding the EPDDv3. This 11-item EPDDv3 was evaluated in a third wave of interviews in Japan and the fourth wave of interviews in the US. During Wave 3 of the Japanese interviews, translation modifications were made to 12 items and wording modifications were made to two items. During Wave 4 of the US interviews, all patients confirmed comprehension of all items. After the final wave of interviews, the EPDDv3 was sent for translation and linguistic validation into 17 languages (including Japanese). The EPDDv3 is displayed in Table 8, and a comparison of the items included in the EPDDv1 and EPDDv2 is displayed in Table 9.

**Table 8:** Full Version of the EPDDv3

#	Item / instruction	Response options	Logic
*EPDDv3 (core)*			
1	The first questions are about vaginal bleeding or spotting that could happen during your period or between periods		
1a	During the past 24 h, did you have any vaginal bleeding or spotting?	Checklist: Yes or No	If no, go to Section 2
1b	During the past 24 h, have you been on your period?	Checklist: Yes or No	n/a
2	The next question is about pain. Please be sure to think only about pain related to your endometriosis when answering this question.		
2a	During the past 24 h, at its worst, how severe was your endometriosis-related pain?	Numeric rating scale: 0 (No pain) to 10 (worst pain imaginable)	n/a
3	The next questions are about sexual activity and pain. When answering, think only about pain that occurs during vaginal penetration.		
3a	During the past 24 h, did you engage in any sexual activity that involved full vaginal penetration?	Checklist: Yes or No	If no, go to item 3c
3b	During the past 24 h, at its worst, how would you rate your level (degree) of pain felt during or following vaginal penetration?	Numeric rating scale: 0 (No pain) to 10 (worst pain imaginable)	n/a *Note: Question only asked if answer to question 3a is yes*
*EPDDv3 (extended)*			
3c	During the past 24 h, did you choose not to have any sexual activity that involved full vaginal penetration for any reason, even though you had the chance?	Checklist: Yes or No	If no, go to item 3e
3d	During the past 24 h, did you choose not to have any sexual activity that involved full vaginal penetration because of your endometriosis?	Checklist: Yes or No	n/a *Note: Question only asked if answer to question 3c is yes*
3e	During the past 24 h, did your desire toward sexual intimacy decrease due to your endometriosis?	Checklist: Yes or No	n/a
4	The following questions are about your daily activities during the past 24 h.		
4a	During the past 24 h, how difficult has it been to do your daily activities?	Numeric rating scale: 0 (not difficult) to 10 (extremely difficult)	n/a
5	On the next screens you will be asked to record the medication you took for your endometriosis-related pain.		
5a	During the past 24 h, did you use your rescue medication for your endometriosis-related pain?	Checklist: Yes or No	If yes, go to item 5b If no, end
5b	During the past 24 h, how many tablets of your rescue medication did you use?	Spinner range 0–20	n/a *Note: Screen only displayed if answer to question 5a is yes*

**Table 9:** Items of the EPDD v1 and v2

EPDDv1	EPDDv2
Pelvic pain (including dysmenorrhea & NMPP)	
During the past 24 h, did you have any vaginal bleeding or spotting?	During the past 24 h, did you have *any* vaginal bleeding or spotting?
During the past 24 h, have you been menstruating (vaginal bleeding or spotting during your period)?	During the past 24 h, have you been menstruating (vaginal bleeding or spotting during your period)?
During the past 24 h, at its worst, how severe was your endometriosis-related pain?	During the past 24 h, at its worst, how severe was your endometriosis-related pain?
–	Was the pain you experienced during the past 24 h: 1. Stable (constant pain that doesn’t change much, and never pain free). 2. Intermittent (I feel pain sometimes, but other times I’m pain-free) 3. Variable (“background” pain all the time, but sometimes pain is worse than at other times)
Dyspareunia and sexual activity	
During the past 24 h, did you have sexual intercourse or engage in any sexual activity that involved full vaginal penetration?	During the past 24 h, did you have sexual intercourse or engage in any *other* sexual activity that involved full vaginal penetration?
During the past 24 h, at its worst, how would you rate your level (degree) of pain at the entrance of the vagina during or following vaginal penetration?	During the past 24 h, at its worst, how would you rate your level (degree) of pain at the entrance of the vagina during or following vaginal penetration?
During the past 24 h, at its worst, how would you rate your level (degree) of pain felt DEEP in your vagina during or following DEEP vaginal penetration?	During the past 24 h, at its worst, how would you rate your level (degree) of pain felt DEEP in your *body* during or following DEEP vaginal penetration?
–	During the past 24 h, did you avoid sexual intercourse?
During the past 24 h, did you avoid sexual intercourse because of your endometriosis?	*You said you avoided sexual intercourse in the past 24 h.* During the past 24 h, did you avoid sexual intercourse because of your endometriosis?
–	During the past 24 h, did you feel a decreased interest in sexual intimacy?
Pain during defecation	
–	During the past 24 h, did you have one or more bowel movements?
–	During the past 24 h, at its worst, how severe was the pain when you had a bowel movement?
Bleeding	
You said you had been menstruating during the past 24 h. On average, how heavy was this bleeding or spotting compared to your usual menstruation?	You said you had been menstruating during the past 24 h. On average, how heavy was this bleeding or spotting compared to your usual menstruation?
During the past 24 h, did you have any vaginal bleeding or spotting related to sexual activity that involved full vaginal penetration?	During the past 24 h, did you have any vaginal bleeding or spotting related to sexual activity that involved full vaginal penetration?
You said you had vaginal bleeding or spotting related to sexual activity during the past 24 h. On average, how heavy was this bleeding or spotting compared to your usual menstruation?	You said you had vaginal bleeding or spotting related to sexual activity during the past 24 h. On average, how heavy was this bleeding or spotting compared to your usual menstruation?
–	During the past 24 h, did you have any vaginal bleeding or spotting related to one or more bowel movements?
–	You said you had vaginal bleeding or spotting related to one or more bowel movements during the past 24 h. On average, how heavy was this bleeding or spotting compared to your usual menstruation?
During the past 24 h, did you have any vaginal bleeding or spotting, not related to menstruation? (Please do not include vaginal bleeding or spotting related to sexual activity that involved full vaginal penetration)	During the past 24 h, did you have any other vaginal bleeding or spotting, not related to menstruation? (Please do not include vaginal bleeding or spotting related to *bowel movements or* sexual activity that involved full vaginal penetration)
You said you had *other* vaginal bleeding or spotting during the past 24 h that was not due to your period or sexual activity or bowel movements. On average, how heavy was this bleeding or spotting compared to your usual menstruation?	You said you had other vaginal bleeding or spotting during the past 24 h that was not due to your period or sexual activity or bowel movements. On average, how heavy was this bleeding or spotting compared to your usual menstruation?
Hot flashes	
During the past 24 h, did you have any hot flashes?	During the past 24 h, did you have any hot flashes?
During the past 24 h, how many hot flashes did you have?	During the past 24 h, how many hot flashes did you have?
Daily activities	
During the past 24 h, how much did your endometriosis-related pain interfere with your daily activities?	During the past 24 h, how *difficult has it been to do your daily activities*?
–	During the past 24 h, have you walked?
–	During the past 24 h, how difficult has it been to walk?
–	During the past 24 h, have you exercised?
–	During the past 24 h, how difficult has it been to exercise?
–	During the past 24 h, have you done any social activities?
–	During the past 24 h, how difficult has it been to do social activities?
–	During the past 24 h, have you done any leisure activities?
–	During the past 24 h, how difficult has it been to do leisure activities?
–	During the past 24 h, have you done any household activities?
–	During the past 24 h, how difficult has it been to do household activities?
–	During the past 24 h, have you done any work or school activities?
–	During the past 24 h, how difficult has it been to do work or school activities?
–	Do you have a spouse/partner?
–	During the past 24 h, how difficult has your relationships been with your partner?
–	During the past 24 h, how difficult have your relationships been with other important people in your life, e.g., family, friends, people at work?
–	During the past 24 h, how difficult has it been to wash or dress yourself?
–	During the past 24 h, how difficult has it been to fall asleep?
–	During the past 24 h, how difficult has it been to stay asleep?
Rescue medication/protection	
During the past 24 h, how many sanitary products (panty liners, pads or tampons) did you use for any type of vaginal bleeding?	During the past 24 h, how many sanitary products (panty liners, pads or tampons) did you use for any type of vaginal bleeding?
During the past 24 h, did you use your rescue medication for your endometriosis-related pain?	During the past 24 h, did you use your rescue medication for your endometriosis-related pain?
During the past 24 h, how many tablets of your rescue medication did you use?	During the past 24 h, how many tablets of your rescue medication did you use?

## Discussion

In the absence of a fit-for-purpose PRO for the measurement of endometriosis-related pain, we developed an instrument to support evaluation of biopharmaceutical products and to measure key signs and symptoms associated with endometriosis. To accomplish this, a conceptual model of endometriosis and the EPDD was developed on the basis of three sources of evidence: a structured literature review, clinical expert opinion, and qualitative patient concept elicitation interviews. In addition, translation and linguistic validation and patient cognitive interviews facilitated the refinement of the items to ensure patient comprehension and understanding, as well as the ability to respond to items as intended. Through these exercises, the items of the EPDD were revised and refined to ensure relevance to patient pain experience as well as comprehension by patients as intended. Translation and testing in Japanese patients allowed for development of a harmonized instrument.

The EPDD resulting from this content validity work consists of five core items relating to dysmenorrhea, NMPP, and dyspareunia, and six extended items relating to sexual activity, daily activities, and use of pain medication, and will serve as a daily, electronic, patient-reported measure of endometriosis-related pain. In particular, the instrument was designed and developed for the purpose of detecting a treatment response in the context of drug development. Content validity of the EPDD was demonstrated via a rigorous process consisting of several waves of cognitive interviews. In addition, the translatability assessment confirmed its utility in 17 languages, allowing for the potential use of the EPDD in global clinical trials in endometriosis. The 17 languages included in the translatability assessment were chosen based on anticipated countries that would be selected for enrollment in future phase 3 clinical trials. If any of those countries/languages are selected, then a full translation and linguistic validation process would be conducted for each language. The phase 2 TERRA study, a double-blind, randomized, parallel-group, placebo-controlled clinical trial, used the EPDD to assess the efficacy of a GnRH receptor antagonist, ASP1707, in women with endometriosis-associated pelvic pain. To determine a clinically meaningful threshold of improvement, as measured by the EPDD, anchor-based analyses of the degrees of improvement in endometriosis-related pain on the Patient Global Impression of Change, the Brief Pain Inventory, the modified Biberoglu and Behrman, and the European quality of life 5-dimension 5-level scale were performed using the EPDDv1. Results suggested that an improvement of 60% (for overall pelvic pain and NMPP) and 70% (for dysmenorrhea), as measured by the EPDD, would be considered clinically meaningful (TERRA; NCT01767090). The successful completion of this study, which achieved its primary endpoint of reducing endometriosis-associated pelvic pain after 12 weeks of treatment, demonstrates the internal validity and reliability of the EPDDv1 in a clinical trial context.

The PRO reported here has three key advantages over existing measures. First, to our knowledge, the EPDD is the only PRO for endometriosis-related pain that was developed in accordance with guidance set forth by the EMA and FDA. Second, the electronic format allows for convenient, real-time completion of the instrument by the patient, without the requirement for involvement of investigative research site staff. Third, the requirement for daily entry ensures accurate data collection and precludes inaccuracies associated with lengthy recall periods. Conversely, limitations of the EPDD are noteworthy and should be considered. As the instrument was designed specifically for use in clinical trials for the assessment of patient response to treatment, important items relating to patient personal experience with endometriosis (ie, work impairment, hot flashes, impact on sleep) were removed from the final version. In addition, the EPDD does not address pain during defecation or exercise, or other non-pain symptoms that are important to patients, such as bloating, dizziness, nausea, vomiting, difficulties with physical functioning, work/school limitations, relationship interference, or emotional functioning (although many of these were measured in the interim EPDDv2). Together, these characteristics limit the use of the EPDD within clinical practice; however, opportunities exist for expansion of the EPDD to broaden its clinical application.

Although the EPDDv3 can be considered content valid, the remaining psychometric properties of the instrument, including reliability, construct validity, and ability to detect change have not been established. Such psychometric data and quantitative validation can be obtained through the administration of the EPDD with other validated PRO measures (eg, Patient Global Impression of Change) in a longitudinal intervention study, and are required for endorsement (eg, FDA) of the EPDD as fit-for-purpose for use in clinical trials of endometriosis interventions.

## Conclusions

The EPDD is a PRO for the evaluation of endometriosis-related pain. The EPDD represents an important step in providing an effective PRO to evaluate pain associated with endometriosis and its related impacts on patients.

## Abbreviations

ADL: activities of daily living
CHMP: Committee for Medicinal Products for Human Use
CPSSS: Composite Pelvic Signs and Symptoms Score
EHP: Endometriosis Health Profile
EMA: European Medicines Agency
EPBD: Endometriosis Pain and Bleeding Diary
EPDD: Endometriosis Pain Daily Diary
FDA: Food and Drug Administration
NMPP: non-menstrual pelvic pain
PRO: patient-reported outcome
US: United States
